# An inguinal hernia revealing an advanced stage gastric cancer in a young patient: A case report

**DOI:** 10.1016/j.amsu.2022.103974

**Published:** 2022-06-09

**Authors:** Muhamad Zakaria Brimo Alsaman, Lina ghabreau, Zainab Zeino, Ziad Alahmad, Maysam Attar, Seba Haboush, Vairy Rezkallah, Ahmad Darwish, Mazen Mohammad

**Affiliations:** a Faculty of Medicine, University of Aleppo, Aleppo, Syria; b Department of Pathology, Aleppo University Hospital, Aleppo, Syria; c Department of Opthalmology, Abd Al Wahab Agha Hospital, Aleppo, Syria; d Department of Pathology, Tishreen Hospital, Damascus, Syria; e Department of General Surgery, Abd Al Wahab Agha Hospital, Aleppo, Syria

**Keywords:** Young, Adenocarcinoma, Gastric, Cancer, Case report

## Abstract

**Introduction:**

Gastric cancer (GC) is the fifth most common cancer and the fourth leading cause of death. It is much more common in advanced age and it is rare among the youngest patients (under 45 years of age).

**Case presentation:**

we report an unusual presentation of advanced gastric adenocarcinoma in 39-year-old man, who presented to our hospital with inguinal hernia without obvious gastrointestinal symptoms. He had strong family history of cancer, heavy smoking habit and weight loss. The intra-operative procedure identified a cyanotic separate spermatocele which was confirmed by the urologist. During investigation we found multiple liver metastasis in abdominal CT and advanced gastric adenocarcinoma from gastric biopsy and metastasis in spermatic cord sample and peritoneum sample of poorly differentiated adenocarcinoma.

**Conclusion:**

Although stomach adenocarcinoma is extremely rare in young patient but it should be kept in mind of physicians as a possible diagnosis if there are many risk factors.

## Highlights

• Gastric adenocarcinoma is rare among the youngers.
• It mainly presents with epigastric pain, vomiting, dysphagia and loss of appetite.
• We report advanced gastric adenocarcinoma, without obvious gastrointestinal symptoms.

## 1 Introduction

Gastric cancer is the fourth cause of cancer death with 769 000 deaths in 2020 [1].

The vast majority of patients are older than 45 age at diagnosis and it is rare among the youngest patients (under 45 years of age) [2]. On the contrary, some studies showed that there are increasing in gastric cancer incidence rate among young patients without clear reasons [3,4]. Therefore, there is growing interest in reporting gastric cancer in young patients.

Unfortunately, a very large number of patients diagnosed at advanced stage due to late symptoms, so most of them will die within 12 months [2]. Weight loss, anorexia, nausea, abdominal pain, or dysphagia are the most common symptoms of stomach cancer, presence of gastrointestinal symptoms is related to survival and risk of death [5,6].

Here we report an unusual case of inguinal hernia revealing an advanced stage gastric cancer in 39-year-old man without obvious digestive symptoms using SCARE reporting guidelines [14].

## 2 Case presentation

A 39-year-old man, previously healthy, presented to our hospital complaining of a 3-month history of swelling in the right inguinal region, loss of appetite and weight loss. He did not complain of dysphagia or abdominal pain.

A detailed history revealed that the patient had a 20-years history of heavy cigarette smoking (The average: 20 cigarettes per day) and his father and cousin died because of laryngeal cancer. He did not tell any previous illnesses or admission to hospitals.

The clinical examination showed a soft abdomen with bulge in the right inguinal region which increased in size with each coughing episode. There were no signs of liver dysfunction and non-palpable lymph nodes. Laboratory studies was in normal limits.

The suspicion of inguinal herniation led us to perform a surgical intervention.

**Fig. 1:**
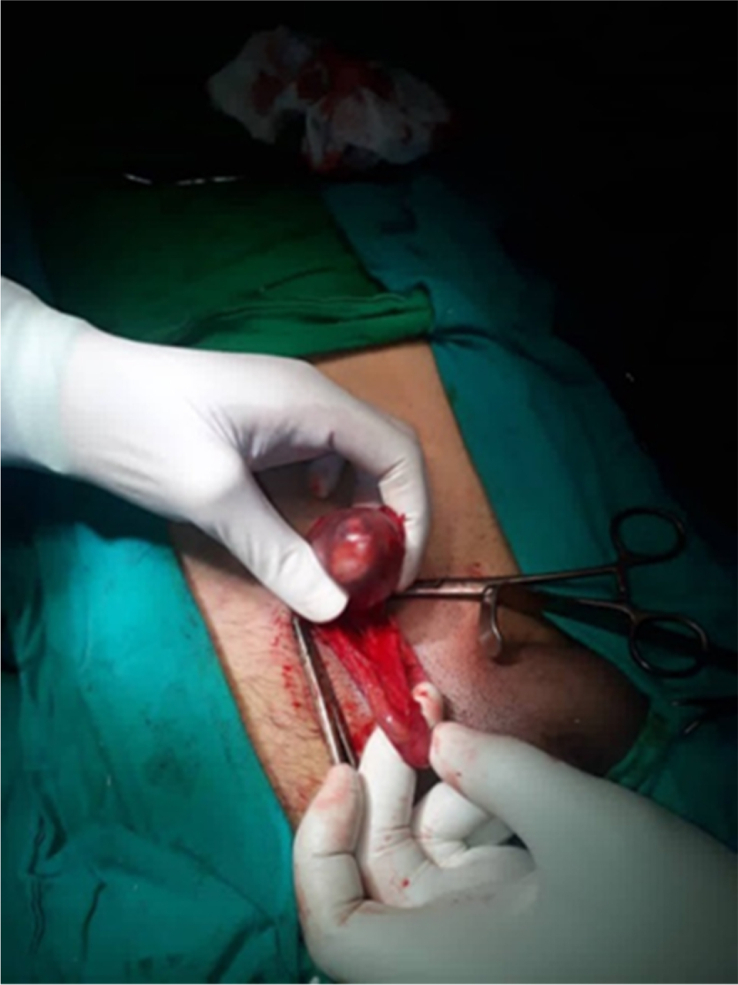
Intraoperative procedure showed a cyanotic separate spermatocele.

Surprisingly, the intra-operative procedure for hernia repair identified a cyanotic separate spermatocele (Fig. 1) which was confirmed by the urologist. It was sent to pathologist for examination.

**Fig. 2:**
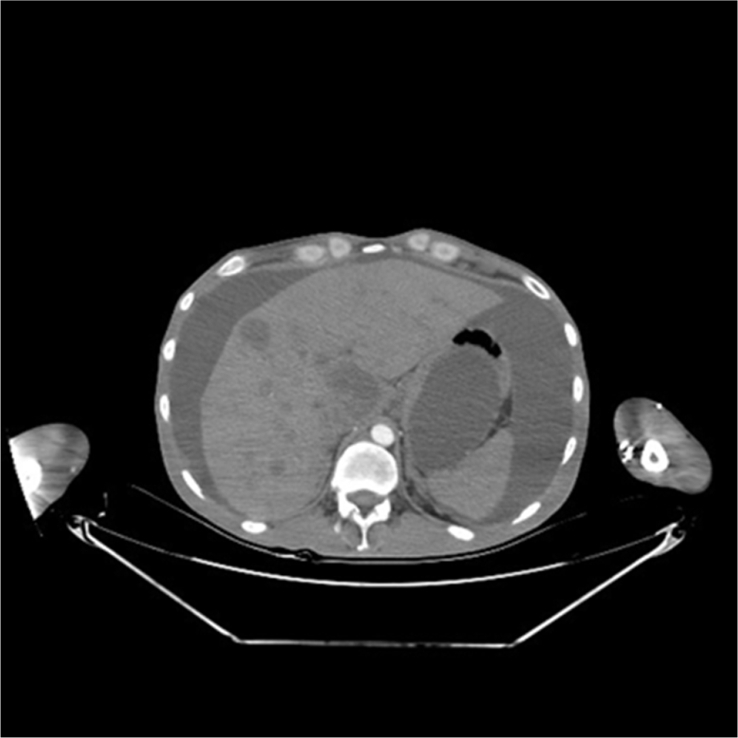
Abdominal ct scan showed multipe metastasis in liver and free fluid around the liver and spleen.

Three days later, the patient presented with an enlarged abdomen which was a high gradient ascites. It was imperative to do an abdomen computed tomography (CT) which showed multiple liver metastasis (Fig. 2).

An upper and lower endoscopy was obtained to find metastasis source. Biopsy sample was obtained from stomach wall during endoscopy and sent to pathology.

**Fig. 3:**
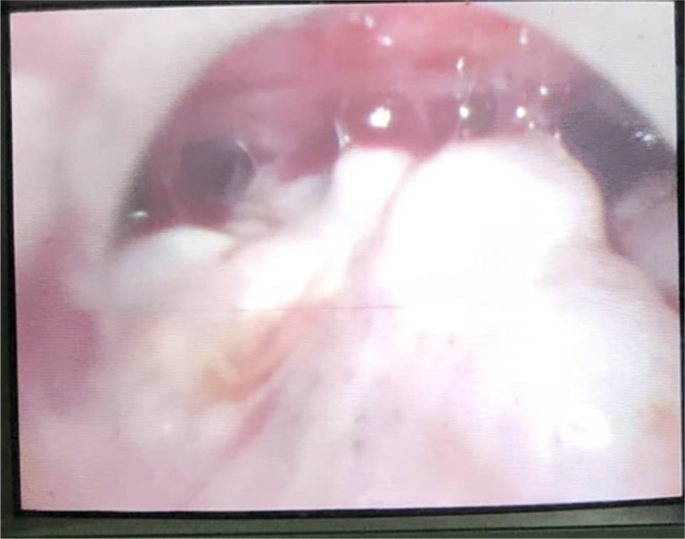
Peritoneal metastasis during laparoscopy.

An urgent diagnostic laparoscopy was performed. Laparoscopy revealed multiple adhesions involving bowel and peritoneal metastasis (Fig. 3) which was biopsied and sent for pathological investigation.

**Fig. 4:**
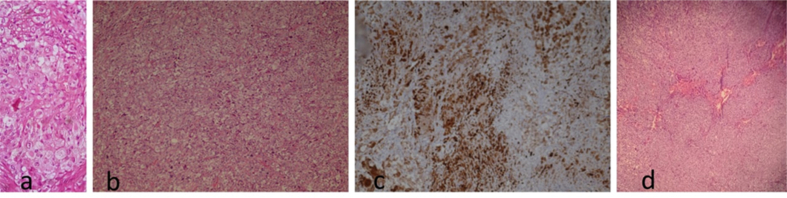
Histopathology examination of samples: (a) From gastric wall biopsy revealed proliferation of malignant epithelial cells composed of sheets and few glandular formations with hyperchromatic nuclei, prominent nucleoli and bizarre mitosis. (b) From the spermatic cord metastasis that revealed malignant epithelial proliferation mad up solid sheets and nests of round to oval tumor cells, showing oval hyperchromatic nuclei with prominent nucleoli and high mitotic figures. Some of tumor cells had signet ring-like appearance. (c) Immunohistochemical stains: CK7: positive, CK20, PLAP,CDx2: negative. (d) From peritoneal metastasis revealed malignant epithelial proliferation composed of solid sheets of round to oval tumor cells, that had vesicular nuclei, prominent nucleoli and some cells had signet ring-like appearance.

Pathology studies revealed advanced gastric adenocarcinoma from gastric biopsy and metastasis in spermatic cord sample and peritoneum sample of poorly differentiated adenocarcinoma (Fig. 4).

The patient's nutritional and clinical situation were not improved due to the advanced stage of the tumor. The patient died within one week of the initial diagnosis.

## 3 Discussion

Gastric cancer is the fifth most common cancer after cancers of the lung, breast, colorectum, and prostate and the fourth leading cause of carcinoma death [1].It occurs in the ages between 50_70 years, and young patients are less likely to get GC, but more aggressive and associated with poor prognosis [2,7].

Even advanced gastric cancer case in young patient was reported previously in our country but it is not endemic [8]. Therefore, reporting gastric carcinoma in young patient help us to understand the relation between age demographics and these cases.

Gastric adenocarcinoma has two different pathological types: intestinal and diffuse, each of them has different appearances, pathogenesis, and genetic profiles. For instance, the intestinal type is well differentiated and includes tubular and glandular elements. The diffuse type is undifferentiated shows poorly cohesive single cells without gland formation [2].

It may present with epigastric pain, vomiting, dysphagia, loss of appetite, upper gastrointestinal bleeding and weight loss [6].

Malignant ascites is very rare complication of gastric cancer. It occurs in late cases and associated with poor prognosis [11].

There are many gastric cancer cases reported in young patients manifested with gastrointestinal symptoms such as; dysphagia, vomiting and hematemesis [[8], [9], [10]].

What distinguishes our case that the patient was diagnosed with advanced gastric adenocarcinoma, although his first complaint was right inguinal hernia without obvious gastrointestinal symptoms.

Many risk factors associated with gastric adenocarcinoma development, such as male sex, advanced age, positive family history, smoking, overdose of alcohol, *Helicobacter pylori* and Epstein–Barr virus (EBV) infections, atrophic gastritis and diet such as chili peppers and salty food. In addition, blood group A and hereditary syndromes such as Li-Fraumeni syndrome, Lynch syndrome, Peutz-Jeghers syndrome increase risk of gastric cancer [2,12].

According to Pisanu et al. who did retrospective cohort study in young patients to determinate the risk factors of GC; younger patients showed a statistically significant higher risk of having a diffuse histological type of gastric carcinoma and *H. pylori* infection [6].

Presence of strong family history of cancer, heavy smoking habit and weight loss in our patient should raise the suspicion of cancer, even patient's history was not, that should direct us to do more than routine investigations.

Gastric cancer stage plays an important role in determination of prognosis.

In general, young or elderly patients have poor prognosis compared to middle-aged patients [7,13].

## 4 Conclusion

Although stomach adenocarcinoma is extremely rare in young patient but it should be kept in mind of physicians as a possible diagnosis if there are many risk factors.Therefore, doctors should not neglect any symptoms or risk factor and take a detailed clinical story.

## Please state any conflicts of interest

The authors declare no conflict of interest.

## Please state any sources of funding for your research

This research was not funded.

## Ethical approval

Not applicable.

## Sources of funding

This research was not funded.

## Author contribution

MZBA, MM. Performed the current surgery: MM. Writing the manuscript, analysis and interpretation of Data: MZBA, ZZ, ZA, MA, SH. Interpretation and providing pathological data: LG, VR, AD. Critical Revision of the paper: MZBA, LG, ZZ, ZA, MA, SH, VR, AD, MM. All authors read and approved the final version of manuscript.

## Consent

A written informed consent was obtained from the patient's wife for publication of this case report and accompanying images.

## Registration of research studies

• Clinicaltrials.gov – for all human studies – free
• Chinese Clinical Trial Registry chictr.org.cn – for all human studies - free
• Researchregistry.com – for all human studies – charge
• ISRCTN.com – for all human studies – charge
• There are many national registries approved by the UN that can be found here

## Elsevier does not support or endorse any registry

1. Name of the registry:
2. Unique identifying number or registration ID:
3. Hyperlink to your specific registration (must be publicly accessible and will be checked):

## Guarantor

Dr. Mazen Mohammad.

## Declaration of competing interest

The authors declare no conflict of interest.
