# Supplementary material for: An inguinal hernia revealing an advanced stage gastric cancer in a young patient: A case report
Source: Ann Med Surg (Lond). 2022 Jun 9;79:103974. doi: 10.1016/j.amsu.2022.103974 (PMC9289344; doi:10.1016/j.amsu.2022.103974)
Supplement: Multimedia component 1 [file mmc1.docx]

| **SCARE Checklist** | | | |
| --- | --- | --- | --- |
| **Topic** | **Item** | **Checklist item description** | **Page Number** |
| **Title** | **1** | **Unusual case of advanced gastric adenocarcinoma in young patient. A case report** | 1 |
| **Key Words** | **2** | young, adenocarcinoma, gastric, cancer, Case Report | 1 |
| **Abstract** | **3a** | Gastric cancer (GC) is the fifth most common cancer and the fourth leading cause of death. It is much more common in advanced age and it is rare among the youngest patients (under 45 years of age). | 1 |
|  | **3b** | 39-year-old man, who presented to our hospital with inguinal hernia without obvious gastrointestinal symptoms. He had strong family history of cancer, heavy smoking habit and weight loss. |  |
|  | **3c** | multiple liver metastasis in abdominal CT and advanced gastric adenocarcinoma from gastric biopsy and metastasis in spermatic cord sample and peritoneum sample of poorly differentiated adenocarcinoma. |  |
|  | **3d** | Although stomach adenocarcinoma is extremely rare in young patient but it should be kept in mind of physicians as a possible diagnosis if there are many risk factors. |  |
| **Introduction** | **4** | Weight loss, anorexia, nausea, abdominal pain, or dysphagia are the most common symptoms of stomach cancer, presence of gastrointestinal symptoms is related to survival and risk of death (5,6).  Here we report an unusual case of advanced gastric adenocarcinoma in 39-year-old man without obvious digestive symptoms using SCARE reporting guidelines (14). | 2 |
| **Patient Information** | **5a** | A 39-year-old man, previously healthy, | **2** |
|  | **5b** | presented to our hospital complaining of a 3-month history of swelling in the right inguinal region, loss of appetite and weight loss. |  |
|  | **5c** | He did not tell any previous illnesses or admission to hospitals. |  |
|  | **5d** | the patient had a 20-years history of heavy cigarette smoking (The average: 20 cigarettes per day) and his father and cousin died because of laryngeal cancer |  |
| **Clinical Findings** | **6** | The clinical examination showed a soft abdomen with bulge in the right inguinal region which increased in size with each coughing episode. There were no signs of liver dysfunction and non-palpable lymph nodes. Laboratory studies was in normal limits. | 2 |
| **Timeline** | **7** | A 39-year-old man, previously healthy, presented to our hospital complaining of a 3-month history of swelling in the right inguinal region, loss of appetite and weight loss.  the patient had a 20-years history of heavy cigarette smoking (The average: 20 cigarettes per day) and his father and cousin died because of laryngeal cancer. He did not tell any previous illnesses or admission to hospitals.  The clinical examination showed a soft abdomen with bulge in the right inguinal region which increased in size with each coughing episode. There were no signs of liver dysfunction and non-palpable lymph nodes. Laboratory studies was in normal limits.  The suspicion of inguinal herniation led us to perform a surgical intervention.  Surprisingly, the intra-operative procedure for hernia repair identified a cyanotic separate spermatocele (Figure.1) which was confirmed by the urologist. It was sent to pathologist because of malignancy suspicion.  Three days later, the patient presented with an enlarged abdomen which was a high gradient ascites. It was imperative to do an abdomen computed tomography (CT) which showed multiple liver metastasis (Figure.2)  An upper and lower endoscopy was obtained to find metastasis source. Biopsy sample was obtained from stomach wall during endoscopy and sent to pathology.  An urgent diagnostic laparoscopy was performed. Laparoscopy revealed multiple adhesions involving bowel and peritoneal metastasis (Figure.3) which was biopsied and sent for pathological investigation.  Pathology studies revealed advanced gastric adenocarcinoma from gastric biopsy and metastasis in spermatic cord sample and peritoneum sample of poorly differentiated adenocarcinoma (Figure.4).  The patient's nutritional and clinical situation were not improved due to the advanced stage of the tumor. The patient died within one week of the initial diagnosis. | 2+3 |
| **Diagnostic Assessment** | **8a** | the intra-operative procedure for hernia repair identified a cyanotic separate spermatocele (Figure.1).  an abdomen computed tomography (CT) which showed multiple liver metastasis (Figure.2)  Laparoscopy revealed multiple adhesions involving bowel and peritoneal metastasis (Figure.3).  Pathology studies revealed advanced gastric adenocarcinoma from gastric biopsy and metastasis in spermatic cord sample and peritoneum sample of poorly differentiated adenocarcinoma (Figure.4). | 2 |
|  | **8b** | There was no impediment or diagnostic challenges. |  |
|  | **8c** | The suspicion of inguinal herniation led us to perform a surgical intervention. |  |
|  | **8d** | advanced gastric adenocarcinoma from gastric biopsy and metastasis in spermatic cord sample and peritoneum sample. |  |
| **Therapeutic Intervention** | **9a** | The patient previously healthy, also He did not tell any previous illnesses or admission to hospitals. | 2+3 |
|  | **9b** | Not mentioned |  |
|  | **9c** | Not mentioned |  |
|  | **9d** | A general surgeon |  |
|  | **9e** | Not mentioned |  |
|  | **9f** | prompt pain control, assessment of the surgical site |  |
| **Follow-up and**  **Outcomes** | **10a** | The patient died within one week of the initial diagnosis. | 3 |
|  | **10b** | Pathology studies revealed advanced gastric adenocarcinoma from gastric biopsy and metastasis in spermatic cord sample and peritoneum sample of poorly differentiated adenocarcinoma (Figure.4). |  |
|  | **10c** | The patient tolerate the procedure |  |
|  | **10d** | (2+3) the Case presentation section |  |
| **Discussion** | **11a** | (Even advanced gastric cancer case in young patient was reported previously in our country but it is not endemic (8). Therefore, reporting gastric carcinoma in young patient help us to understand the relation between age demographics and these cases.  (What distinguishes our case that the patient was diagnosed with advanced gastric adenocarcinoma, although his first complaint was right inguinal hernia without obvious gastrointestinal symptoms. | 3+4 |
|  | **11b** | (Gastric cancer is the fifth most common cancer after cancers of the lung, breast, colorectum, and prostate and the fourth leading cause of carcinoma death (1). It occurs in the ages between 50_70 years, and young patients are less likely to get GC, but more aggressive and associated with poor prognosis (2,7).  (Gastric adenocarcinoma has two different pathological types: intestinal and diffuse, each of them has different appearances, pathogenesis, and genetic profiles. For instance, the intestinal type is well differentiated and includes tubular and glandular elements. The diffuse type is undifferentiated shows poorly cohesive single cells without gland formation (2).  (Malignant ascites is very rare complication of gastric cancer. It occurs in late cases and associated with poor prognosis (11).  (According to Pisanu et al. who did retrospective cohort study in young patients to determinate the risk factors of GC; younger patients showed a statistically significant higher risk of having a diffuse histological type of gastric carcinoma and H. pylori infection (6). |  |
|  | **11c** | (2+3) the sections (Case presentation + Discussion) |  |
|  | **11d** | (Presence of strong family history of cancer, heavy smoking habit and weight loss in our patient should raise the suspicion of cancer, even patient’s history was not, that should direct us to do more than routine investigations. )  (Although stomach adenocarcinoma is extremely rare in young patient but it should be kept in mind of physicians as a possible diagnosis if there are many risk factors. Therefore, doctors should not neglect any symptoms or risk factor and take a detailed clinical story. ) |  |
| **Patient Perspective** | **12** | the patient was satisfied after surgery but he died within one week . | 3 |
| **Informed Consent** | **13** | Consent was taken from the patient’s wife |  |
| **Additional Information** | **14** | No conflicts of Interest and There are no sources of funding. |  |
